# Picky Eating Is Associated with Lower Nutrient Intakes from Children’s Home-Packed School Lunches

**DOI:** 10.3390/nu13061759

**Published:** 2021-05-21

**Authors:** Kellseigh Gan, Carly Tithecott, Lisa Neilson, Jamie A. Seabrook, Paula Dworatzek

**Affiliations:** 1 School of Food and Nutritional Sciences, Brescia University College at Western University, 1285 Western Road, London, ON N6G 1H2, Canada; kellseighgan@gmail.com (K.G.); ctithec2@gmail.com (C.T.); lisajneilson@gmail.com (L.N.); jseabro2@uwo.ca (J.A.S.); 2 Department of Paediatrics, Schulich School of Medicine & Dentistry, Western University, 1151 Richmond Street, London, ON N6A 3K7, Canada; 3 Department of Epidemiology & Biostatistics, Western University, 1151 Richmond Street, London, ON N6A 3K7, Canada; 4 Human Environments Analysis Laboratory, Western University, 1151 Richmond Street, London, ON N6A 3K7, Canada; 5 Children′s Health Research Institute, 800 Commissioners Road East, London, ON N6C 2V5, Canada; 6 Lawson Health Research Institute, 750 Base Line Road East, Suite 300, London, ON N6C 2R5, Canada; 7 Schulich Interfaculty Program in Public Health, Schulich School of Medicine & Dentistry, Western University, 1465 Richmond St., London, ON N6G 2M1, Canada

**Keywords:** picky eating, fussy eating, food neophobia, packed lunch intake, children’s dietary intake, elementary school

## Abstract

The objective was to assess the relationship between children’s picky eating (PE) status and nutrient intake from home-packed school lunches. The lunches of 321 students, aged 7–10 years, were quantified via cross-sectional direct observation. Children were classified as having PE (*n* = 155) or not (non-PE; *n* = 166) based on food neophobia scores and parental perceptions of PE. The PE group consumed significantly less protein, folate, magnesium, potassium, zinc, and vitamins B1, B2, B3, B6, D, and E than the non-PE group; however, both groups consumed amounts exceeding Dietary Reference Intakes (DRIs) for protein, carbohydrates, sugar, sodium, iron, and vitamins B1, B2, B3, B6, B12, and C. Conversely, both groups consumed amounts significantly lower than DRIs for calcium, fibre, folate, magnesium, potassium, zinc, and vitamins A, D, E, and K. The PE group ate significantly less meat and alternatives, vegetables and fruit, and fruit than the non-PE group, and did not meet any of Canada’s Food Guide (2007) recommendations. The non-PE group met recommendations for meat and alternatives only. PE impacts the dietary intake of children’s home-packed lunches; however, many packed lunches were of low nutritional quality. Focus should be placed on provision of nutritionally complete school lunches for all children.

## 1. Introduction

When a child’s eating behaviours are restrictive and invariable, it can be classified as food neophobia (FN) or picky eating (PE). FN is the fear or avoidance of novel foods, possibly evolved from an innate fear of unknown foods to prevent the ingestion of harmful substances [1,2]. PE extends beyond FN, with continued rejection of foods even after the initial novelty is gone [1]. Children with PE typically consume a limited variety of foods, flavours, and textures [1,3,4]. Packing school lunches can be particularly challenging when the child has picky or fussy eating tendencies [5,6]. Caregivers report children’s preferences as a barrier to packing school lunches and may pack less nutritious items that the child likes to ensure that they will eat [5,6,7]. This is concerning in Canada as there is no national or provincial school food program and most children must bring a home-packed lunch to school every day [8].

Despite some characteristic differences between PE and FN, there are similarities in their implications on dietary intake and body weight. Several studies have found associations between PE, and sometimes FN, and lower body mass index (BMI) [4,9,10,11,12], while others found no difference in weight status, especially for FN [12,13,14]. Of those that found differences in BMI, some report that children with PE are more likely to be underweight [9,10,12,15], and/or less likely to be overweight [4,9,11,12]. Furthermore, children with PE display poor diet quality and limited variety [12,16,17]. Specifically, they have lower vegetable and fruit consumption [3,12,15,18,19], and lower energy intake overall [20], while having a higher intake of sweetened foods [15,17]. Similarly, children who display FN may have less variety in their diets [21], consume fewer vegetables and fruit [1,4,12,22,23,24], calories [4], and protein [18,22] than other children. They may also have higher intakes of snack foods [12,22,23] and saturated fat [21]. One study reported that PE is associated with continued low intake of vegetables and fruit, whole grains, and high intakes of snack foods, sugar-sweetened beverages, and fast food into young adulthood [25]. 

Caregivers perceive that the foods children consume at school and at home are of high importance [5,7]. At home, parents respond to PE with strategies such as encouraging or even pressuring the child to try foods, role modelling, rewards/punishments, avoiding the food to avoid waste, repeated exposure to foods, allowing the child to go without eating, accepting that the child will select the foods that they want to eat, and permitting a separate meal prepared by the child or a parent [26,27,28]. Conversely, when the child is at school, parents may perceive less control over their child’s intake, and fear that the child will not eat at all if their preferences are unavailable [5,7,29]. Thus, parents may include items of poorer nutritional quality in packed lunches to ensure that their child will eat what is packed in order to avoid hunger and/or waste [5,7,29]. 

PE is not an uncommon phenomenon; it may affect 13–36% of toddlers [13,16,20,30], and 30–50% of children [12,16,20]. Children at higher risk of PE may include those whose families have lower household incomes, lower levels of education, and those who were born with complications or at a lower birth weight [13,15,20,30]. Particularly severe and persistent selective/restrictive eating behaviours are of concern [27] and may even result in malnutrition and poor growth. Children who present with Avoidant/Restrictive Food Intake Disorder, a recent addition to the DSM-V, have often experienced PE since early childhood [31]. Understanding how these behaviours present and evaluating their potential effects or outcomes is important for developing preventative interventions.

While FN and PE have been shown to negatively affect children’s food intake, previous studies often have small sample sizes or focus on toddler-aged children [3,4,17,19,21,22]. Of the population-level studies comparing intake of children with PE to those without, the focus is often on the assessment of food groups/categories vs. nutrients [12,15,25], or there was no statistical comparison of nutrient intakes to recommendations [13]. No population studies to our knowledge have assessed nutrient intake of school-aged children by PE classification and compared intakes to reference standards/recommendations. The purpose of this study was to evaluate the relationship between PE and the quality of children’s dietary intake at school, when consuming home-packed lunches. Specifically, the primary objective was to assess caloric intake of home-packed lunches in those with or without PE at the population level. Secondary objectives included the comparison of nutrient and food group intakes in the two groups as well as to reference standards/recommendations.

## 2. Materials and Methods

### 2.1. Study Population and Design

This study conducted a secondary analysis of data available from another study [32]. Elementary school principals in the Thames Valley District School Board, who agreed to host this study, facilitated recruitment of grade 3 and 4 students (aged 7 to 10 years). A cross-sectional, direct observation method was employed to record all visible food and beverage items consumed by each student during a typical school day. One observation day per student was considered adequate to assess group intake only [33]. Methodological details on the direct observation dietary assessment method are provided in previous publications [32,34].

A trained research assistant measured the height and weight of each participant to calculate BMI. The World Health Organization Growth Charts were used to determine the corresponding gender-specific BMI-for-age percentile and weight category [35], primary measures used to assess weight status, as they account for growth and changes in body fat and are more appropriate for use in children than BMI [36].

### 2.2. Parental Survey

Surveys completed by a parent/caregiver of each participating student obtained information on children’s FN and PE behaviours, as well as possible hindrances to packing a lunch for consumption at school. FN and PE were measured using two methods: 1) a modified Child Food Neophobia Scale (CFNS), and 2) a single five-point scale to assess parental perception of PE. The CFNS scale is based on the Food Neophobia Scale (FNS) originally developed by Pliner and Hobden [37], which was later adapted for children by removing questions related to ethnic foods, restaurants, and dinner parties [22,23,38]. The resultant CFNS was highly correlated with behavioural outcomes when children were presented with ten familiar foods and ten novel foods [38]. For the purposes of this study, the CFNS was condensed from six items to four to promote a high response rate and remain relevant to the context of home-packed school lunches [22]. The four CFNS items used related to a child’s willingness to try unfamiliar foods, a child’s fear of unfamiliar foods, the degree to which a child is particular about foods consumed, and the degree to which a child will eat almost anything (reverse scored). The questions that were removed are unlikely to affect overall scoring as they re-frame items already addressed (i.e., child does not trust new food, child is constantly sampling new food (reverse scored)). Each item was answered on a five-point strongly disagree to strongly agree scale. Possible scores ranged from four to twenty, with higher numbers indicating higher FN. The modified four-question version of the CFNS used in this study resulted in high internal consistency (Cronbach’s alpha = 0.91).

The second assessment of PE in the survey was a single five-point item to assess parental perception of the child’s PE status. Many authors have noted that the literature lacks a consistent definition and measurement tool for PE [1,3,9,17,39]; however, caregiver response to a single question has been shown to predict PE behaviour [40,41,42]. Despite this, at least one review has indicated that the use of one question to assess PE is a concern [39], thus we assessed the relationship between the 4-item CFNS and the PE question. These two techniques demonstrated high internal consistency (Cronbach’s alpha = 0.93). Furthermore, when parental responses to the PE item were collapsed into three groups (agree, neutral, and disagree) there was a statistically significant difference in CFNS scores between groups, as determined by one-way analysis of variance (ANOVA) (F(2317) = 219.31, *p* < 0.001). Children whose parents agreed with the PE item had higher mean CFNS scores (15.35 ± 2.91), compared to children whose parents disagreed (7.96 ± 2.47, *p* < 0.001) or had a neutral response (11.97 ± 3.29, *p* < 0.001). Based on these scores, children with a CFNS score of ≥12 were assigned to the PE group. The term PE was selected over FN because caregivers typically identify restrictive eating behaviours as “picky” or “fussy” eating, and because PE is more comprehensive than FN. Additionally, only 2 of the 4 CFNS items specifically related to a ‘fear of new foods’ (i.e., a child’s willingness to try unfamiliar foods, and a child’s fear of unfamiliar foods), while the remaining two could be categorized as picky eating (i.e., the degree to which a child is particular about foods consumed, and the degree to which a child will eat almost anything (reverse scored)).

### 2.3. Statistical Analyses

All recorded food and beverage items were entered into ESHA Food Processor software (version 10.12; ESHA Research, Salem, OR) and coded with Canadian Nutrient File or, when necessary, U.S. Department of Agriculture items. Data were analyzed using the Statistical Package for the Social Sciences (SPSS; IBM Corp., Version 22.0, Armonk, NY). For all analyses, a *p*-value < 0.05 was considered statistically significant.

To assess demographic differences between children classified with or without PE, continuous and categorical demographic variables were compared by independent-samples t-tests and chi-square tests, respectively. The normality of each nutrient distribution was assessed with the Shapiro–Wilk test. All nutrient distributions in this sample were non-normally distributed, with the exception of percentage of Calories from fat (data not shown). Thus, the non-parametric Mann–Whitney U test was used to compare the median for calories and nutrients by PE vs. non-PE status (except percent Calories from fat, which was compared via the independent-samples *t*-test). 

The comparison of PE vs. non-PE status alone is insufficient to assess nutritional adequacy, because the non-PE group may have excessive or inadequate dietary intakes [43]; therefore, the median nutrient intakes of both groups were compared to available reference standards via the one-sample Wilcoxon signed rank test (except percent Calories from fat, which was compared by the one-sample t-test). As Canada does not have established recommendations for home-packed school meals, Dietary Reference Intakes (DRIs) were used as reference standards [44]. Specifically, Estimated Average Requirements (EARs) were preferred for comparison to nutrient intakes, as these are deemed appropriate for evaluation of groups [45,46]. When EARs were unavailable, adequate intakes (AIs) were used [44]. The DRIs for children aged 9–13 years old were deemed appropriate based on the mean age of the sample (i.e., 9.12 ± 0.63 years). Reference standards were divided by three to represent one-third of daily intake, to reflect the American Dietetic Association’s recommendation for children to consume one-third of daily intake in programs lasting 4–7 h [47] and scientific findings that children consume approximately one-third of their daily calories at school [48].

Alternative reference standards were necessary when both EAR and AI values were unavailable for specific nutrients. The reference standards used for total energy intake were Estimated Energy Requirements (EERs) for low-active, 9-year-old, male and female children [49]. The low activity level was selected because it is estimated that Canadian children spend 60% of their waking hours participating in sedentary behaviours [50]. The reference standard for sugar was calculated based on Health Canada’s percent daily value for sugar of 20%, reflecting a total daily sugar intake of 100 g (400 kcal) based on a 2000 kilocalorie (kcal) diet [51]. This recommendation was adjusted to 20% of the energy recommended by the EER for children [49]. The reference standard for total fat intake was the acceptable macronutrient distribution range (AMDR) for 4–18 year olds [49]. The saturated fat reference was The Dietary Guidelines for Americans (2010) recommendation for less than 10% of daily energy intake [52]. As the reference standards for total fat and saturated fat were measured as percentage of total Calories, it was not necessary to divide them by three.

Recorded food and beverage items were classified into the four food groups defined by the 2007 Eating Well with Canada’s Food Guide (CFG): vegetables and fruit, grain products, milk and alternatives, and meat and alternatives [53]. Food items were also classified separately in the following food categories, deemed relevant for home-packed lunches: fruits, vegetables, 100% fruit/vegetable juice, sugar-sweetened beverages, and snacks [32,54,55,56,57,58]. The snack category was defined as a non-entrée, non-beverage, non-fresh fruit or vegetable, sweet or savoury item, packaged for consumption in one sitting. One snack serving was based on the typical size of pre-packaged snack items found at grocery retailers (20–35 g or 100–120 mL). One serving of a sugar-sweetened beverage was 125 mL for comparison to a CFG serving of 100% fruit juice. Food group intake distributions were negatively skewed due to the presence of many zero values; thus, the non-parametric Mann–Whitney U test compared median portions of each food group consumed. The one-sample Wilcoxon signed rank test compared intake to the lower end of CFG recommendations, based on the assumption that meeting the minimum recommendation is sufficient for most children.

Finally, an open-ended question included in the parental survey asked, “Please describe anything that hinders your ability to provide a packed lunch for your child,” to assess potential barriers for packing school lunches. Two researchers independently analyzed and coded the qualitative responses that related to PE specifically, to identify common themes.

## 3. Results

A total of 321 grade 3 and 4 students, aged 7–10 years, were observed in 19 elementary schools in the Thames Valley District School Board in Ontario, Canada. The majority (97.2%) of caregiver respondents who completed the survey were parents of the participants. A total of 155 students (48.3%) were classified as having PE based on their CFNS score; demographic variables were not significantly different between those with and without PE (Table 1). 

The BMI-for-age median percentiles of those with PE was not significantly different from those without PE, and frequencies in BMI-for-age classifications were not different between groups (Table 1). Similarly, the total school day caloric intake was not significantly different for those with or without PE (for both males and females) (Table 2). The PE group, however, consumed significantly less of 12 of 24 nutrients compared to the non-PE group (i.e., protein, fibre (in males only), folate, magnesium, potassium, vitamins B1, B2, B3, B6, D, and E, and zinc). Median intakes of calcium, fibre, folate, magnesium, potassium, vitamins A, D, E, and K, and zinc were significantly lower than reference standards for both groups. Intakes of protein, carbohydrates, sugar, sodium, iron, and vitamins B1, B2, B3, and C were greater than reference standards in both groups. Four of the nutrients that were consumed in amounts exceeding recommendations (iron, sodium, vitamins B3, and C) have tolerable upper intake levels (ULs) [44]. Iron and vitamin C intakes were significantly lower than ULs (*p* < 0.001) among both PE and non-PE groups, while B3 intake was equivalent to the UL among those with PE (*p* = 0.06), and greater than the UL among those with no PE (*p* = 0.01). Similarly, sodium intake was equal to the UL among the PE group (*p* = 0.65), and significantly greater than the UL among the non-PE group (*p* = 0.001).

When compared to recommendations, most nutrients were either over- or under-consumed by both groups, with the exception of percent Calories from saturated fat, vitamin B6, and vitamin B12 (Table 3). The intake of these three nutrients did not differ from reference standards among those with PE, suggesting adequacy. Among those without PE, intakes of vitamins B6 and B12 were significantly greater than DRIs, and percent Calories from saturated fat was significantly lower than the reference standard. Median intakes of energy and percent Calories from fat did not significantly differ from the reference standards for both groups, indicating that population-level intake was neither excessive nor insufficient. In summary, nutrients of concern for inadequacy for both groups included calcium, fibre, folate, magnesium, potassium, vitamins A, D, E, and K, and zinc, with the PE group consuming even lower amounts for fibre (males), folate, magnesium, potassium, vitamins E and D, and zinc. Nutrients of concern for excessive intake in both groups included sugar and sodium, with no differences by PE status. 

When considering food groups/categories, the PE group consumed significantly less vegetables and fruit, meat and alternatives, and fruit than the non-PE group (Table 4). There were no significant differences between groups in the amounts of grain products, milk and alternatives, vegetables, fruit juice, sugar-sweetened beverages, or snacks consumed from home-packed lunches. Compared to servings recommended by CFG, intake of all four food groups was insufficient among the PE group, while the non-PE group consumed sufficient servings of meat and alternatives only. Neither group consumed enough of the vegetables and fruit food group at school to meet one-third of CFG recommendations; however, when vegetables and fruit were analyzed separately, the non-PE group consumed significantly more fruit than the PE group.

Table 5 summarizes the PE themes identified from the responses to the open-ended question: “Please describe anything else which hinders your ability to provide a packed lunch for your child”. Specific hindrances identified included aversions, limited variety, changing preferences, allergy policies, sensitivities to temperature and texture, neophobia, and fear child will go hungry were all identified as PE-related hindrances for parents when packing school lunches.

## 4. Discussion

The prevalence of PE among this sample of 7–10-year-old children appears high at 48.3%; however, the average age of this sample is older than other studies which focused on toddler-aged children [13,20,30]. A longitudinal study of PE found that with increasing age, new cases of PE decreases, but overall prevalence increases [16], suggesting that PE may accumulate in a cohort of children, resulting in a relatively high prevalence by 7–10 years. It is also possible that packing and consuming a school lunch that is eaten away from home, without refrigeration or re-heating equipment, may pose additional difficulties for children who have PE tendencies. Furthermore, parents may be more limited in their strategies to deal with their child’s PE when the child is eating away from home and may be more aware of the challenges in doing so [5,6,7].

The BMI-for-age percentiles for PE and non-PE groups did not differ significantly. These results contradict some previous findings that those with PE tend to have lower BMIs and are classified as underweight more than those without PE [4,10,11,59]. This could be due in some cases to the lower ages of children in previous studies. As children increase in age, parents may develop strategies to ensure the child is eating sufficient calories for growth. Our data support this concept as there was no significant difference in caloric intake from home-packed lunches. PE may significantly compromise nutrient intake in environments where food is scarce; however, in the modern industrialized food environment, a wide variety of palatable, energy-dense foods are readily and cheaply available [4]. Availability of these options may allow those with PE to match energy consumed by non-PE children to achieve similar BMI-for-age percentiles.

The PE group ate significantly fewer servings of meat and alternatives, vegetables and fruit, and fruit than the non-PE group. This is consistent with one population-level study, which found that 14-month-old children with PE consumed less vegetables and fish than the non-PE group [15]. They also found that the 14-month-old PE group consumed more savoury snacks and sweets [15], consumption of which is atypical at this age. This was not a finding of the current study, likely because participants were older and of an age that is associated with excess consumption of sweet and/or savoury processed snacks [60]. Food group intakes were not compared to recommendations in previous studies [3,13,15,19,21,22], so this study was unique in finding that packed lunch intake of children with PE did not meet one-third of CFG’s recommended daily servings in any of the four food groups. The non-PE group met recommendations for meat and alternatives only. One reason participants failed to meet CFG recommendations may be that this sample had a mean age of 9.12 ± 0.63 years, which is on the lower end of the 9–13-year-old category for the 2007 CFG. Another possible explanation may be a high intake of items not represented by food groups in CFG. In 2004, Canadians aged 2–18 years consumed 55.1% of their daily energy from ultra-processed foods such as soft drinks, fruit drinks and juices, confectionary, fast food, and breakfast cereals [60]. Although items such as cereals, fruit juice, and some fast foods may be included in the 2007 CFG [53], other fortified processed snack items such as brownies and cookies can contribute to meeting DRIs, but are not accounted for in CFG. Fortified processed snack items are convenient for caregivers to pack in lunches, and they may feel more confident that their child with PE will consume these items [6,7]. Packed lunches for all children, and particularly those with PE, may contain fortified processed foods, but lack enough whole, nutrient-dense foods to meet CFG recommendations. This could explain why those with PE in the current study met and exceeded DRIs for some nutrients, without meeting any of the CFG recommendations. It is noteworthy that a few findings from this analysis may have been impacted if the 2019 CFG had been available at the time of this study [61]. The milk and alternatives and meat and alternatives food groups were combined to form one “protein” food category. Children with PE may have been more likely to meet this protein food category recommendation compared to the previous food groups, given that they significantly exceeded the DRI for protein. The 2019 CFG also recommended a larger proportion of intake from vegetables and fruits, and 100% fruit juice no longer contributed to this food category. In this study, even with 100% fruit juice contributing to vegetables and fruits, both the PE and non-PE groups consumed significantly less than the CFG 2007 recommendation. Compared to the new, higher recommendation, and with 100% fruit juice removed, both PE and non-PE groups would likely show an even greater insufficiency. Overall, failure to meet CFG recommendations is consistent with a study of Canadian adolescents, which found that only 5% of males and 7% of females met the 2007 CFG recommendations [62].

In addition to the differences in food group intake, the PE group also consumed significantly less protein, fibre, folate, magnesium, potassium, vitamins B1, B2, B3, B6, D, and E, and zinc than the non-PE group. Other studies that investigated differences in the intake of specific nutrients between those with and without PE did not have consistent findings, and often had smaller sample sizes with less comprehensive nutrient analysis. For example, one study found no dietary differences between those with and without PE [17], while others found lower energy intake [22], or lower intake of fibre and folate [4] among those with PE. Li et al. evaluated a population-level sample of toddlers with comprehensive nutrient analysis, and found at 6–11 months of age, those with PE consumed significantly less vitamin A, B1, B2, B6, and C, but at 12–23 months nutrient intake did not differ between those with and without PE [13]. As the current study compared the observed lunch intake of school-aged children with and without PE at the population level, perhaps differences could be observed that were not detected by smaller studies, or possibly home-packed lunches present more challenges than meals eaten at home, for both parents and their children with PE.

Many of the smaller studies that measured the association of PE and dietary intake compared a group of children with PE to a group with normal eating behaviours, without comparison to recommendations [3,15,18,22]. This is not an accurate assessment of nutritional adequacy if the group with normal eating behaviours also does not meet recommendations. The Canadian Community Health Survey (2.2) found that the Canadian adolescent population does not meet dietary reference standards for several nutrients [43]. When the population as a whole does not meet recommendations, deviation from “normal eaters” may not reflect nutritional inadequacy or excess. For example, in the present study, the PE group consumed significantly less protein than the non-PE group, which might be construed as inadequacy, as was the case in previous studies [18,22]; however, upon statistical comparison to reference intakes, protein intake was not deficient in our study. Caregivers may also have a perception of protein inadequacy when they experience feeding difficulties or compare the intake of their PE child to other children [13,26]. Parents of those with PE in this study expressed difficulty providing a main source of protein in their child’s lunch due to aversions to protein sources and school allergy policies (peanut-free); however, both the PE and non-PE groups appeared to meet protein DRIs in their packed lunches. Thus, perceptions of insufficient intake may be unfounded.

Compared to dietary reference standards, nutrient intakes were consistently excessive or insufficient among both the PE and non-PE groups; however, the PE group consumed even lower amounts of fibre (males), folate, magnesium, potassium, vitamins E and D, and zinc. This aligns with the significantly lower intake of vegetables and fruit in this group and seems to be the most significant concern for those with PE identified in this study. Nevertheless, despite these differences in nutrient intakes, overall issues in packed-lunch intake are similar among all children, regardless of PE status. Both the PE and non-PE groups consumed insufficient amounts of calcium, fibre, folate, magnesium, potassium, zinc, and vitamins A, D, E, and K. These results are consistent with the Canadian Community Health Survey, which indicated that Canadian adolescents under-consumed calcium, fibre, magnesium, potassium, and vitamins A and D [43], and a smaller study that found children under-consumed vitamin E, folate, calcium, and zinc [21].

Intakes of both the PE and non-PE groups exceeded recommendations for protein, carbohydrates, sugar, sodium, iron, and vitamins B1, B2, B3, B6, B12, and C. Several of these nutrients are among those that may or must be added to fortified foods in Canada [63], which could explain why they are consumed in excess, but generally without concern, among children. For example, vitamin C is often added to juices and fruit drinks [63], which are marketed for inclusion in school lunches. While fortified juices can contribute vitamin C to the diet, they are not the preferred source of nutrients compared to whole fruits, which provide additional nutrients, phytochemicals, and dietary fibre [64]. In addition, white and enriched flour products must be fortified with vitamins B1, B2, B3, and iron in Canada [63]. Canada’s Food Guide (2007) recommends that half of grain products consumed be whole grain to ensure adequate intakes of fibre and magnesium [64], although whole grain products are not fortified [63]. The 2019 CFG recommends consuming about a quarter of the total diet as whole grains, and does not include a recommendation for fortified, refined grain products [61]. If children consume refined grain products and snack items, their intakes of the nutrients included in fortification may be higher while intakes of magnesium and fibre, present in whole grain products, may remain low. Additionally, ready-to-eat cereals are often voluntarily fortified with vitamins B1, B3, and B6, and iron, among others [63]. Falciglia et al. found that fewer children met dietary recommendations when ready-to-eat cereals were excluded from analysis [21], implying that these products help children meet some dietary recommendations. Frequent consumption of ready-to-eat cereals is associated with decreased risk of micronutrient deficiency, but is also associated with increased risk of high sugar intake [65]; thus, they are not the preferred source of nutrients compared to whole grain, minimally processed sources of B-vitamins and iron without added sugar. Fortified foods have been reported as the main source of nutrients in children’s diets [66], and the prevalence of these processed items in packed school lunches may contribute to nutrients consumed in adequate or excess amounts, but they may not be the preferred source of nutrients.

The Dietary Guidelines for Americans (2010) recommends saturated fat intake remain below 10% of total energy [52]. In this sample, the non-PE group met this recommendation and the PE group intake was not statistically different from this recommended maximum. The Canadian Community Health Survey (2.2) reported that adolescents over-consumed saturated fat, as well as sodium and sugar [43], which may be found in the snack items and sugar-sweetened beverages commonly packed in school lunches. Both the PE and non-PE groups in this study consumed amounts of sugar and sodium significantly above reference values, and the non-PE group significantly exceeded the UL for sodium. Excess consumption of these nutrients among the PE and non-PE group may be a concern for long-term health, as negative dietary habits formed in childhood may endure throughout the lifespan [67]. It is known that excessive consumption of saturated fat, sodium and sugar increases the risk of numerous chronic diseases, such as diabetes, heart disease, high blood pressure, high blood cholesterol, stroke, obesity, and cancer [68,69,70]. Continuing to consume these nutrients in excess may put both the PE and non-PE groups at risk of adverse health conditions.

Responses from the parental survey indicated that a child’s PE behaviours interfere with their parent’s ability to pack school lunches. Limited and fluctuating preferences, fear of new foods, sensitivities to texture and temperature, and specific aversions all restricted what parents could pack in their child’s lunch. Some parent responses indicated concern that their child would go hungry at school if they packed foods the child would not eat and may have chosen to pack less nutritious items if they were confident their child would consume those. Participants in other studies similarly reported what they packed in their child’s lunch was influenced by the child’s preferences, even if that meant packing less healthy items [6,7].

Parents further identified factors related to the school environment that limited what they were able to pack, such as the inability to refrigerate and re-heat food, and school allergy policies. Sabrina’s Law was enacted in January 2006, and required Ontario school boards to create and maintain school-wide allergy policies, including strategies to reduce the risk of exposure in common areas to food items with the potential to trigger anaphylactic reactions (e.g., peanuts) [71]. Parents in this study reported that allergy policies limited the peanut and nut products they were able to send in their child’s packed school lunch. This has importance as these are healthy plant-based proteins they knew their child would eat. Parents also reported struggling with packing lunches appropriate for a child’s sensitivity to temperature, as there was no way to keep foods refrigerated or re-heat them at school. Parents in other studies have also cited food safety concerns with leaving foods at room-temperature, particularly in the summer, as a barrier to what they could pack [7]. The literature suggests that PE may also be related to tactile sensitivity [72], so barriers to temperature control in the school environment may further limit what children with PE will consume from packed lunches compared to at-home meals. Overall, caregivers of children with PE may struggle to pack nutritious school lunches when faced with barriers related to the child’s preferences as well as the school environment.

Contributions of this study include its evaluation of the nutritional intake of children with PE at the population level and, unlike previous population studies, analysis of specific nutrients and statistical comparison of intake to dietary reference standards [13,15]. The method of direct observation is an effective measure of dietary intake because it is not dependent on children’s memory and provides unbiased information on actual intake [73,74]. Other methods such as pictorial 24 h recall, food frequency questionnaires, and diet records have underestimated and overestimated intake [33]. Additionally, the use of both quantitative and qualitative data, known as mixed methods, strengthens research findings [75]. Finally, this sample size is larger than that of many other studies that examined PE or FN and dietary intake [3,4,16,17,19,21,22].

Despite the strengths in study design, the following limitations should be considered. The absence of a universal definition and measurement tool for PE must be taken into account [1,3,9,17,39]. Some studies have highlighted the lack of variety in the diets of children with PE [16,17,21]; however, this was not assessed in the current study. Another factor to consider is that intake was compared to one-third of dietary reference standards, based on findings that school-aged children consume approximately one-third of their daily calories at school [48]. It is possible, however, that children, as a group, can overcome the inadequacies in intake during the school day resulting in 24 h intake that is adequate. This seems unlikely, however, given the population-level studies that have shown that children and adolescents in Canada have inadequate intakes [43,60,62].

## 5. Conclusions

Compared to nutrient reference standards, children classified as having PE consumed some nutrients in excess, and others in insufficient quantities; however, children without PE demonstrated a similar pattern and tended to over- or under-consume the same nutrients. Nevertheless, children who have PE consumed significantly fewer desirable nutrients than those who did not have PE. The PE group also ate significantly fewer servings from several food groups compared to the non-PE group and failed to meet CFG recommendations for any food groups, while the non-PE group met recommendations for one food group. The packed lunches of those with PE were of poor nutritional quality compared to both the non-PE group and reference standards, although the packed lunch consumption of all children failed to meet several recommendations.

Although PE impacts children’s dietary intake from home-packed lunches at school, many of the deficiencies that exist among this population are also found in general populations of children who consume home-packed lunches at school [43,54,55,56,57,58]. A better understanding of how PE influences the intake of specific nutrients in children can provide insight into areas of concern. Interventions targeted to improve PE behaviours should consider the particular concerns related to home-packed school lunches (i.e., advocacy for refrigeration and reheating equipment). It is also possible that curricular/in-class learning could focus on food exposures, food sensory experiences, and food skills as this would be beneficial for those with and without PE. Perhaps the most impactful way to promote population-wide interventions that encourage the consumption of unprocessed, nutrient-dense foods at school would be the implementation of a universal school lunch program. This may help to improve intake among children with picky eating, as well as improving the nutritional intake of all Canadian school children.

## Figures and Tables

**Table 1:** Demographic characteristics by picky eating status.

Characteristics		Total	PE ^1^	Non-PE	*p*-Value ^2^
Number of Participants, % (*n*)		100 (321)	48.3 (155)	51.7 (166)	
Sex, % (*n*)					0.54
	female	49.8 (160)	51.6 (80)	48.2 (80)	
	male	50.2 (161)	48.4 (75)	51.8 (86)	
Grade, % (*n*)					0.1
	three	53.6 (172)	49.0 (76)	57.8 (96)	
	four	46.4 (149)	51.0 (79)	42.2 (70)	
Age (years), mean (SD)		9.12 (0.63)	9.14 (0.64)	9.10 (0.62)	0.50
School Location, % (*n*)					0.53
	rural	41.4 (133)	43.2 (67)	39.8 (66)	
	urban	58.6 (188)	56.8 (88)	60.2 (100)	
BMI for Age, Percentile (kg/m^2^ %ile; mean (SD))		63.54 (31.90)	60.30 (33.23)	66.57 (30.39)	0.08
Parental Age (years)^3^, % (*n*)					0.58
	20–29	7.1 (22)	7.5 (11)	6.7 (11)	
	30–39	51.4 (160)	48.3 (71)	54.3 (89)	
	≥40	41.5 (129)	44.2 (65)	39.0 (64)	
Parent Educational Attainment ^3,4^, % (*n*)					0.79
	less than post-secondary	28.0 (90)	27.1 (42)	28.9 (48)	
	post-secondary	67.0 (215)	67.1 (104)	66.9 (111)	
Household Income ($), mean (SD) ^3,5^		54 821.81 (45 245.02)	58 016.97 (45 952.68)	51 838.38 (44 505.50)	0.22
BMI-for-Age, Weight Classification ^6^, % (*n*)					0.41
	Underweight	2.2 (7)	3.2 (5)	1.2 (2)	
	Normal	60.1 (193)	61.9 (96)	58.4 (97)	
	Overweight	18.1 (58)	18.1 (28)	18.1 (30)	
	Obesity	19.6 (63)	16.8 (26)	22.3 (37)	

PE, Picky eating; non-PE, non-picky eating; SD, standard deviation; BMI, body mass index. ^1^ Picky eating defined as food neophobia score ≥12. ^2^ Continuous variables with mean and standard deviation were analyzed by the independent-samples t-test. Categorical variables with percentage and *n* were analyzed by the chi-square test; *p*-value < 0.05 considered significant. ^3^ Total sample size reduced due to missing data as respondents were able to decline to answer or skip questions on the survey. ^4^ Survey responses for educational attainment were collapsed into two groups, less than post-secondary (less than high school, some high school, high school graduate) and post-secondary (college/trade school graduate, university degree). ^5^ Participants reported income within a given range. The midpoint of the range was quantified to calculate an average. ^6^ Weight classification determined by WHO Growth Charts BMI-for-Age Percentile Cutoffs. Underweight (wasting) < 3rd BMI-for-age percentile, normal weight 3rd–85th percentile, overweight > 85th–97th percentile, obesity > 97th percentile.

**Table 2:** Nutrients consumed from home-packed school lunches by picky eating status.

Nutrient	PE ^1^ (*n* = 155) Median Intake (Interquartile Range: Q1, Q3)		Non-PE (*n* = 166) Median Intake (Interquartile Range: Q1, Q3)		*p*-Value ^2^
Energy (kcal)	M: 542.32 ^3^	(413.84, 701.22)	597.48 ^3^	(502.15, 751.99)	0.08
	F: 516.18 ^4^	(387.71, 715.36)	573.06 ^4^	(419.34, 694.13)	0.47
Protein (g)	13.56	(8.67, 20.06)	17.01	(10.79, 23.03)	**0.01**
Calories from Fat ^5^ (%)	27.50	± 10.71	26.96	± 10.57	0.65
Calories from Saturated Fat (%)	9.73	(6.32, 13.49)	9.13	(5.88, 12.19)	0.36
Carbohydrates (g)	87.08	(62.57, 104.67)	93.05	(67.00, 114.12)	0.12
Sugar (g)	39.28	(26.62, 54.50)	41.23	(23.79, 57.95)	0.62
Calcium (mg)	205.96	(99.35, 340.77)	222.00	(127.69, 325.23)	0.52
Fibre (g)	M: 3.80 ^3^	(2.70, 5.25)	5.03 ^3^	(3.26, 6.43)	**0.02**
	F: 3.99^4^	(2.66, 6.06)	4.91 ^4^	(3.47, 6.88)	0.08
Folate DFE (mcg)	57.94	(25.52, 106.28)	73.67	(41.65, 132.81)	**0.001**
Iron (mg)	M: 3.34 ^3^	(1.94, 4.65)	3.81 ^3^	(2.85, 5.41)	0.06
	F: 3.22 ^4^	(1.82, 4.29)	3.55 ^4^	(2.39, 4.83)	0.25
Magnesium (mg)	55.70	(37.55, 76.84)	62.26	(44.41, 86.65)	**0.03**
Potassium (mg)	526.60	(307.63, 731.93)	601.81	(386.74, 760.06)	**0.04**
Sodium (mg)	714.60	(444.01, 1051.40)	828.90	(546.10, 1187.42)	0.06
Vitamin A (RAE)	M: 77.70 ^3^	(16.21, 153.83)	66.05 ^3^	(26.52, 161.96)	0.95
	F: 74.37 ^4^	(19.52, 161.94)	66.78 ^4^	(23.32, 175.08)	0.87
Vitamin B1 (mg)	0.34	(0.17, 0.51)	0.42	(0.27, 0.59)	**<0.01**
Vitamin B2 (mg)	0.38	(0.25, 0.58)	0.44	(0.32, 0.63)	**0.047**
Vitamin B3 NE (mg)	5.84	(3.46, 8.83)	7.01	(4.93, 9.87)	**0.001**
Vitamin B6 (mg)	0.26	(0.17, 0.42)	0.32	(0.19, 0.50)	**0.01**
Vitamin B12 (mcg)	0.50	(0.19, 0.93)	0.58	(0.22, 1.05)	0.18
Vitamin C (mg)	15.19	(5.26, 54.56)	25.04	(8.45, 69.27)	0.08
Vitamin D (mcg)	0.08	(0.00, 0.36)	0.13	(0.03, 0.50)	**0.04**
Vitamin E Alpha-Tocopherol (mg)	0.77	(0.38, 1.45)	0.94	(0.52, 1.61)	**0.04**
Vitamin K (mcg)	5.85	(2.28, 13.18)	6.48	(2.67, 14.30)	0.09
Zinc (mg)	1.71	(1.11, 2.43)	2.10	(1.26, 2.77)	**0.01**

PE, Picky eating; non-PE, non-picky eating; M, males; F, females; RAE, retinol activity equivalents; NE, niacin equivalents. ^1^ Picky eating defined as food neophobia score ≥12. ^2^ Differences in medians were assessed using the Mann–Whitney U test because data were skewed; *p*-value <0.05 considered significant; significant findings are bolded. ^3^ M and F have a different reference amount, intake among males, total males *n* = 161, 75 PE, 86 non-PE. ^4^ M and F have a different reference amount, intake among females, total females *n* = 160, 80 PE, 80 non-PE. ^5^ Nutrient was normally distributed and therefore evaluated with the independent-samples *t*-test, and reported with the means ± standard deviation.

**Table 3:** Nutrient consumption from home-packed school lunches by picky eating status and recommended intakes.

Nutrient	DRI ^1^/3	PE (*n* = 155) Median Intake (Interquartile Range: Q1, Q3)		PE Intake-DRI/3	*p*-Value ^2^	Non-PE (*n* = 166) Median Intake (Interquartile Range: Q1, Q3)		Non-PE Intake-DRI/3	*p*-Value ^2^
*Nutrient Intakes Exceeding Recommendations*									
Protein (g)	6.33	13.56	(8.67, 20.06)	7.23	**<0.001**	17.01	(10.79, 23.03)	10.68	****<0.001****
Carbohydrates (g)	33.33	87.08	(62.57, 104.67)	53.75	**<0.001**	93.05	(67.00, 114.12)	59.72	**<0.001**
Sugar (g) ^3^	M: 29.17 ^4^	39.34	(29.47, 54.45)	10.17	**<0.001**	45.03	(29.55, 58.56)	15.86	**<0.001**
	F: 26.67 ^5^	38.95	(26.17, 56.46)	12.28	**<0.001**	38.27	(18.84, 57.04)	11.60	**<0.001**
Iron (mg)	M: 1.97 ^4^	3.34	(1.94, 4.65)	1.37	**<0.001**	3.81	(2.85, 5.41)	1.84	**<0.001**
	F: 1.90 ^5^	3.22	(1.82, 4.29)	1.32	**<0.001**	3.55	(2.39, 4.83)	1.65	**<0.001**
Sodium (mg) ^6^	500	714.60	(444.01, 1051.40)	214.6	**<0.001**	828.9	(546.10, 1187.42)	328.9	**<0.001**
Vitamin B1 (mg)	0.23	0.34	(0.17, 0.51)	0.11	**<0.001**	0.42	(0.27, 0.59)	0.19	**<0.001**
Vitamin B2 (mg)	0.27	0.38	(0.25, 0.58)	0.11	**<0.001**	0.44	(0.32, 0.63)	0.17	**<0.001**
Vitamin B3 NE (mg)	3	5.84	(3.46, 8.83)	2.84	**<0.001**	7.01	(4.93, 9.87)	4.01	**<0.001**
Vitamin B6 ^7^	0.27					0.32	(0.19, 0.50)	0.05	**<0.001**
Vitamin B12(mcg) ^7^	0.5					0.58	(0.22, 1.05)	0.08	**<0.001**
Vitamin C (mg)	13	15.19	(5.26, 54.56)	2.19	**<0.001**	25.04	(8.45, 69.27)	12.04	**<0.001**
*Nutrient Intakes Meeting Recommendations (i.e., not significantly lower or higher than DRI/3)*									
Energy ^8^ (kcal)	M: 583.33 ^4^	542.32	(413.84, 701.22)	−41.01	0.35	597.48	(502.15, 751.99)	14.15	0.17
	F: 533.33 ^5^	516.18	(387.71, 715.36)	−17.15	0.64	573.06	(419.34, 694.13)	39.73	0.14
Calories from Fat (%) ^9,10^	25–35 ^11^	27.50	± 10.71	Within AMDR		27.96	± 10.57	Within AMDR	
Calories from Saturated Fat (%) ^12^	<10 ^11^	9.73	(6.32, 13.49)	−0.27	0.50				
Vitamin B6 (mg)	0.27	0.26	(0.17, 0.42)	−0.01	0.28				
Vitamin B12 (mcg)	0.5	0.50	(0.19, 0.93)	0.00	0.07				
*Nutrient Intakes Below Recommendations*									
Calories from Saturated Fat (%)	<10% ^11^					9.13	(5.88, 12.19)	−0.87	**0.02**
Calcium (mg)	366.67	205.96	(99.35, 340.77)	−160.71	**<0.001**	222.00	(127.69, 325.23)	−144.67	**<0.001**
Fibre (g) ^6^	M: 10.33 ^4^	3.80	(2.70, 5.25)	−6.53	**<0.001**	5.03	(3.26, 6.43)	−5.30	**<0.001**
	F: 8.67 ^5^	3.99	(2.66, 6.06)	−4.68	**<0.001**	4.91	(3.47, 6.88)	−3.76	**<0.001**
Folate DFE (mcg)	83.33	57.94	(25.52, 106.28)	−25.39	**<0.001**	73.67	(41.65, 132.81)	−9.66	**0.026**
Magnesium (mg)	66.67	55.70	(37.55, 76.84)	−10.97	**0.001**	62.26	(44.41, 86.65)	−4.41	**0.005**
Potassium (mg) ^6^	1500	526.60	(307.63, 731.93)	−973.4	**<0.001**	601.81	(386.74, 760.06)	−898.19	**<0.001**
Vitamin A (RAE)	M: 148.33 ^4^	77.70	(16.21, 153.83)	−70.63	**0.002**	66.05	(26.52, 161.96)	−82.28	**0.001**
	F: 140.00 ^5^	74.37	(19.52, 161.94)	−65.63	**0.002**	66.78	(23.32, 175.08)	−73.22	**0.004**
Vitamin D (mcg)	3.33	0.08	(0.00, 0.36)	−3.25	**<0.001**	0.13	(0.03, 0.50)	−3.20	**<0.001**
Vitamin E Alpha-Tocopherol (mg)	3	0.77	(0.38, 1.45)	−2.23	**<0.001**	0.94	(0.52, 1.61)	−2.06	**<0.001**
Vitamin K (mcg) ^6^	20	5.85	(2.28, 13.18)	−14.15	**<0.001**	6.48	(2.67, 14.30)	−13.52	**<0.001**
Zinc (mg)	2.33	1.71	(1.11, 2.43)	−0.62	**<0.001**	2.10	(1.26, 2.77)	−0.23	**<0.001**

DRI, dietary reference intakes; PE, Picky eating; non-PE, non-picky eating; M, males; F, females; AMDR, acceptable macronutrient distribution range; NE, niacin equivalents; DFE, dietary folate equivalents; RAE, retinol activity equivalents. ^1^ Median intakes were compared to Estimated Average Requirements (EAR), as is appropriate for analysis of group intake. ^2^ One-sample Wilcoxon signed rank test used to compare median to DRI because data were non-normally distributed; *p*-value < 0.05 considered significant; significant findings are bolded. ^3^ Used daily value of 100 g total sugar (or 20% of energy) from 2000 kcal diet; calculated 20% of EER recommendations for each sex and age group; converted to grams of sugar. ^4^ Male dietary recommendation. Total males *n* = 161; 75 PE, 86 non-PE. ^5^ Female dietary recommendation. Total females *n* = 160; 80 PE, 80 non-PE. ^6^ Median intake was compared to adequate intake (AI) value because EAR was unavailable. ^7^ Nutrient intake for PE group indicated below in *Nutrient Intakes Meeting Recommendations*. ^8^ Median energy intake was compared to Estimated Energy Requirement (EER) values by age group for each sex. ^9^ Median intakes were compared to the upper and lower limits where applicable. Median intakes were deemed to be within limits if there was no significant difference from the lower limit or the higher limit. ^10^ Nutrient was normally distributed and therefore presented as mean ± standard deviation. Mean nutrient intake was compared to reference standard with the one-sample t-test. ^11^ Recommendations for fat intake are represented by percentages of total Calories, therefore were not divided by three to establish lunch recommendations. ^12^ Nutrient intake for non-PE group indicated below in *Nutrient Intake Below Recommendations*.

**Table 4:** Food group consumption from home-packed school lunches by PE status compared to CFG 2007 recommendations.

Food Group or Category	Recomm- Endations ^1^/3	PE (*n* = 155) Median Intake (Interquartile Range: Q1, Q3)		PE Intake-CFG	*p*-Value ^2^	Non-PE (*n* = 166) Median Intake (Interquartile Range: Q1, Q3)		Non-PE Intake-CFG	*p*-Value ^2^	*p*-Value ^3^ PE vs. Non-PE
Veg and Fruit	2	0.71	(0.00, 1.41)	−1.29	**<0.001**	1.00	(0.26, 2.00)	−1.00	**<0.001**	**0.001**
Grains	2	1.22	(0.50, 1.98)	−0.78	**<0.001**	1.30	(0.80, 2.00)	−0.70	**<0.001**	0.28
Milk and Alt	1	0.42	(0.00, 0.99)	−0.58	**<0.001**	0.51	(0.00, 0.92)	−0.49	**<0.001**	0.66
Meat and Alt	0.33	0.00 ^4^	(0.00, 0.46)	−0.33	**<0.01**	0.31	(0.00, 0.59)	−0.69	0.72	**<0.01**
Fruit ^5^		0.22	(0.00, 1.00)			0.84	(0.00, 1.14)			**<0.001**
Vegetables ^5^		0.00 ^4^	(0.00, 0.27)			0.00 ^4^	(0.00, 0.50)			0.24
Fruit Juice ^5^		0.00 ^4^	(0.00, 0.00)			0.00 ^4^	(0.00, 0.00)			0.71
SSB ^5^		0.00 ^4^	(0.00, 1.60)			0.00 ^4^	(0.00, 1.60)			0.98
Snacks ^5^		1.96	(1.00, 2.96)			2.00	(1.00, 3.08)			0.67

CFG, Canada’s Food Guide; PE, picky eating; non-PE, non-picky eating; Alt, Alternatives; Veg, Vegetables; M, males; F, females; SSB, sugar-sweetened beverages. ^1^ Number of servings recommended by CFG 2007; used lower end of recommended range to represent minimum servings needed to meet recommendations. ^2^ Median intakes were compared to established reference standards using the one-sample Wilcoxon signed rank test for data non-normally distributed; *p*-value < 0.05 was considered significant; significant findings are bolded. ^3^ Median intakes were compared between PE and non-PE groups using the Mann–Whitney U test for data non-normally distributed; *p*-value < 0.05 was considered significant; significant findings are bolded. ^4^ Median values of zero resulted from many students not consuming the food group/category. ^5^ Food categories do not have established reference standards.

**Table 5:** Barriers related to picky eating that hindered parent’s ability to pack a school lunch for their child ^1^.

Aversions
Protein Sources *“Does not like lunch meat or… milk”* *“My child does not like many meats...”* *“My son can be very picky when it comes to the main source of protein in his lunch”* *“It’s often a challenge to send enough protein…”* Sandwiches *“My daughter does not like sandwiches so I have to be creative…”* *“He will not eat sandwiches because in grade one, he was forced to finish it all, now he will never bring one with him”* *“She won’t eat sandwiches”*
**Limited Variety**
*“His “pickiness” limits the variety of foods I can put in his lunch, therefore, he generally eats the same lunch daily.”* *“Do not know or have ideas of alternative foods that [she] might eat.”*
**Changing Preferences**
*“He goes in cycles on what he likes and doesn’t like.”* *“She goes through phases with cereal, salad and soup.”* *“…randomly decides he no longer likes certain foods in his lunch”*
**Allergy Policies**
*“…not allowed any food with nuts, peanut butter* etc. *Very hard when you have a fussy child and that’s something she loves!”* *“My child likes peanut butter but cannot bring any foods with nuts.”* *“Also not being able to bring nut products.”*
**Sensitivities**
*To Texture* *“Child…only eats certain textures of food.”* *To Temperature* *“She doesn’t like cold food, so I have very limited choices.”* *“…only eats hot food, and he doesn’t: 1. like the look of food in a thermos 2. feel it is kept hot enough in thermos”* *“Not having any way to heat foods.”*
**Neophobia**
*“[He]…does not like to try anything new”* *“My child…hates to try new things”*
**Fear Child Will Go Hungry**
*“…when I pack it sometimes she doesn’t like the stuff I put in it.”* *“If she doesn’t like it or feel like eating it, she won’t”* *“Not having groceries, knowing he may not eat what I pack…”* *“Try to pack lunches that he will eat.”* *“I most likely pack too much and that way she can choose what she wants.”*

^1^ Qualitative responses to “Please describe anything that hinders your ability to provide a packed lunch for your child”.

## Data Availability

The data presented in this study are available on request from the corresponding author.
